# Rationale for MYC imaging and targeting in pancreatic cancer

**DOI:** 10.1186/s13550-021-00843-1

**Published:** 2021-10-12

**Authors:** Günter Schneider, Matthias Wirth, Ulrich Keller, Dieter Saur

**Affiliations:** 1 Medical Clinic and Policlinic II, Klinikum Rechts Der Isar, TU Munich, 81675 Munich, Germany; 2 German Cancer Research Center (DKFZ) and German Cancer Consortium (DKTK), 69120 Heidelberg, Germany; 3 Department of General, Visceral and Pediatric Surgery, University Medical Center Göttingen, 37075 Göttingen, Germany; 4 Department of Hematology, Oncology and Cancer Immunology, Campus Benjamin Franklin, Charité - Universitätsmedizin Berlin, 12203 Berlin, Germany; 5 Max-Delbrück-Center for Molecular Medicine, 13125 Berlin, Germany; 6 Insititute for Translational Cancer Research and Experimental Cancer Therapy, MRI, TU Munich, 81675 Munich, Germany

**Keywords:** Pancreatic cancer, Precision oncology, MYC, Targeted therapies

## Abstract

The incidence and lethality of pancreatic ductal adenocarcinoma (PDAC) will continue to increase in the next decade. For most patients, chemotherapeutic combination therapies remain the standard of care. The development and successful implementation of precision oncology in other gastrointestinal tumor entities point to opportunities also for PDAC. Therefore, markers linked to specific therapeutic responses and important subgroups of the disease are needed. The MYC oncogene is a relevant driver in PDAC and is linked to drug resistance and sensitivity. Here, we update recent insights into MYC biology in PDAC, summarize the connections between MYC and drug responses, and point to an opportunity to image MYC non-invasively. In sum, we propose MYC-associated biology as a basis for the development of concepts for precision oncology in PDAC.

## Pancreatic Ductal Adenocarcinoma (PDAC)

Current estimates show that PDAC will be the second leading cause of cancer-related death reason by 2040 [1]. Together with a dismal prognosis reflected by a 5-year survival rate of 10% [2], these data imply the urgent need to intensify research into the disease. Approximately 85% of patients with PDAC are diagnosed with a locally advanced or disseminated disease that prevents surgical resection. For the small group of patients with a resectable tumor, adjuvant chemotherapy according to a modified regimen with folinic acid, 5‐fluorouracil (5‐FU), irinotecan, and oxaliplatin (mFOLFIRINOX) has been established and has improved overall survival [3]. Furthermore, neoadjuvant therapeutic regimens are in clinical development [3]. For the majority of patients with locally advanced or disseminated disease, systemic chemotherapy with FOLFIRINOX or the combination of nab-paclitaxcel and gemcitabine is used [4]. Although these therapies remain standard of care, overall response rates of only 20–30% and significant toxicities must be considered [4]. Therefore, the development of improved therapies is urgently required.

In contrast with the current “one-size-fits-all” clinical trials, precision oncology based on markers to guide therapy selections has the promises to improve outcomes. Precision oncology is also emerging in gastrointestinal (GI) cancers, and molecular profiling was recently highlighted by the American Society of Clinical Oncology (ASCO) as the advance of the year 2021 for GI cancers [5]. Examples include clinical trials in HER2 positive gastric cancer [6], the Viktory umbrella trial in gastric cancer [7], the approval of targeted therapies for cholangiocarcinomas with *FGFR2* fusions or rearrangements [8], or the use of a triple targeted therapy in *BRAF* mutated colon cancer [9]. Precise therapies have also been shown to affect PDAC patient survival [10]. Thus, further markers that characterize relevant PDAC subtypes, which are associated with specific drug responses, are needed. We propose the concept that the myelocytomatosis oncogene (MYC) is a relevant biomarker for PDAC. This is based on the strong biology and specific phenotypes associated with MYC, the differential drug sensitivity of cancers with deregulated MYC, and the emerging ability to image MYC non-invasively.

## An update of MYC-driven biology in PDAC

*KRAS* mutations occur in over 90% of PDACs [11] and MYC, a basic-helix-loop–helix/leucine zipper transcription factor (TF), has an important function as an integrator of signaling pathways triggered by oncogenic *KRAS* [12]. MYC heterodimers (e.g., MYC/MAX) bind to *cis*-regulatory E-box sequences of numerous genes involved in the regulation of growth, proliferation, or metabolism, thereby serving all hallmark demands of cancer cells [13, 14]. MYC marks an aggressive PDAC subtype, which is supported by the fact that amplifications of *MYC* have been associated with a worse survival of PDAC patients [15]. Pre-clinical experimental evidence underscores that MYC is an essential and non-redundant node of oncogenic signaling and therefore should be an exceptional therapeutic target [16–18]. We have recently summarized PDAC-specific functions of MYC [19–21] and therefore focus here on recent developments. The MYC network is activated in the so-called basal-like subtype, which is the most aggressive subtype and refractory to current therapies [22–26]. *MYC* amplifications occur more frequently in PDAC liver metastases, implicating a role of MYC in this process [27]. Interestingly, metastatic PDAC can be grouped into a low frequency metastasis group (< 10 metastases) and high frequency metastasis (> 10 metastases) group, with the high metastasis frequency group having lower overall survival [28]. Genomic and transcriptomic analyses linked MYC to the high metastasis group [28]. Furthermore, adenosquamous cancers of the pancreas showed a high *MYC* amplification frequency [29].

In addition to its multiple implications in controlling cancer cell biology, MYC`s important role in remodeling the tumor microenvironment (TME) is emerging. The TME is a unique feature of PDAC, evidenced by a prominent desmoplastic reaction that accounts for up to 90% of the tumor mass/volume [30]. Inducing *Myc* together with the *Kras* oncogene in murine in vivo PDAC models has an immediate and profound effect on the TME. This MYC-mediated TME-reprogramming is characterized by the attraction of immune cells such as macrophages, neutrophils, granulocytic myeloid-derived suppressor cells and B-cells, while CD3^+^ T-cells are depleted [31], collectively shaping an immunosuppressive phenotype. Consistently, MYC and BRAF synergistically drive immune-evasion downstream of KRAS [32], supporting a prominent role of MYC in this process. In addition to the immune compartment, MYC instructs the proliferation of fibroblasts and stellate cells, leading to the characteristic tumor desmoplasia [31].

Mechanistically, a link of MYC to immune evasion in PDAC at different levels is described. Double-stranded RNA (dsRNA), derived from intronic inverted repetitive elements, activates the pattern recognition receptor TLR3 and its downstream effector, the serine-threonine kinase TANK-binding kinase 1 (TBK1) [33]. Here, MYC/MIZ1-mediated repression of vesicular transport genes result in decreased dsRNA secretion and consequently decreased activation of TLR3-TBK1-NFκB signaling pathway, which is required for activation of the immune response by controlling the expression of MHC class I antigens [33]. Furthermore, paracrine signals, such as the GAS6–AXL pathway [31] or MYC-dependent repression of the type I interferon pathway [34], account for the TME remodeling. In addition, the folate cycle enzyme methylenetetrahydrofolate dehydrogenase 2 (MTHFD2) was recently demonstrated to facilitate immune escape of PDAC cells. Mechanistically, MTHFD2 promotes the production of uridine diphosphate *N*-acetylglucosamine (UDP-GlcNAc), leading to O-GlcNAcylation of MYC, a post-translational modification (PTM), that results in its stabilization, and the subsequent increased expression of programmed cell death 1 ligand 1 (PD-L1), thereby blocking anti-tumor immune responses [35]. The effects of MYC on the TME have also been connected to the metastatic cascade. It was suggested that a panel of chemokines and cytokines upregulated in MYC overexpressing cells, including MIF, IL24, CCL4, CCL3, CXCL2, or CXCL3, contributes to the recruitment of pro-metastatic tumor-associated macrophages [28].

Importantly, a reciprocal stroma-to-tumor cell signaling is relevant. Here, a pathway is described in which the fibroblast growth factor receptor 1 (FGFR1) activated by FGF1-derived from cancer-associated fibroblasts (CAFs) is augmenting the tumor cell intrinsic MYC signal [36]. Furthermore, JAK1-STAT6-MYC-mediated increase in expression of enolase 1 and hexokinase (HK2)—enzymes involved in glycolysis—is promoted by IL4 and IL13 cytokines present in the TME, revealing the complex modalities of signals integrated by MYC [37]. In sum, MYC not only integrates KRAS-driven cellular signaling, but reciprocally controls communication with the TME to orchestrate the overall tumor biology. Taken together, this novel knowledge of how MYC shapes the TME additionally underscores its value as a therapeutic target and will open opportunities to implement immunotherapies in combination with concepts to block MYC.

## MYC stratifies for therapies

Across all TF and cross-tumor entities, MYC exhibits the most frequent drug interactions [38]. This finding is based on several molecular features. The oncogenic addiction of cancer cells on MYC explains the increased sensitivity of cancer cells to drugs targeting MYC directly or indirectly. Furthermore, MYC-mediated oncogenic stress demands the activation of genes or pathways allowing the cancer cells to cope with. This renders cancer cells with deregulated MYC particularly sensitive to inhibitors of such pathways. The existence of MYC-associated synthetic dosage lethality is well documented by unbiased genetic and pharmacological screening experiments [39] and is relevant in the context of PDAC [21]. Genes allowing to tolerate MYC-induced oncogenic stress may be involved in feedforward regulatory circuits and blocking of such a gene also impacts on MYC expression. Importantly, MYC also marks resistance phenotypes that are currently emerging. Here, we summarize selected connections between MYC and drug sensitivity in PDAC cells.

## Drug resistance and MYC

With regard to resistance phenotypes, MYC has been associated with modulation of the sensitivity of inhibitors of the serine/threonine protein kinase mammalian target of rapamycin (MTOR). MTOR is an important therapeutic target in PDAC, and combination therapies based on MTOR inhibitors (MTORi) are currently under development [40–44]. Genetic gain- and loss-of-function experiments demonstrated that MYC confers resistance to the MTORi INK128 (Sapanisertib) [45], a highly selective ATP-competitive inhibitor of the kinase under clinical development (e.g., NCT02197572, NCT02893930, NCT03430882). The MYC protein has a high turnover regulated by oncogenic signaling that cumulates in post-translational-modifications (PTMs) of the protein. KRAS induces stabilization of MYC through ERK-mediated phosphorylation of serine 62 (S62) [46]. The MYC-phospho-S62 counteracting protein phosphatase 2A (PP2A) de-phosphorylates S62, thereby initiating ubiquitin-dependent degradation of MYC. PP2A is a well-described regulator of MYC protein expression in PDAC [47]. Consequently, small-molecules activators of PP2A, like DT1154 [48], synergize with the MTORi INK128 in PDAC in vitro and in vivo models [45].

Additionally, also secondary resistance phenotypes to targeted therapies may also be mediated by MYC. Interference with the canonical KRAS-MEK-ERK signaling induced downregulation of MYC protein expression. Therefore, MEKi perturbs MYC-directed and pentose phosphate pathway (PPP)-mediated nucleotide synthesis with a subsequent growth arrest. Interestingly, in PDAC cells generated as MEKi resistant, MYC escaped regulation and maintained nucleotide synthesis contributing to the resistance phenotype [49].

MYC-induced resistance occurs not only to targeted therapies, but also with conventional chemotherapeutic agents. MYC has been shown to promote epithelial-neuroendocrine lineage plasticity, a state characterized by the increased expression of neuroendocrine markers, like synaptophysin, and resistance to gemcitabine [50]. Lowering MYC expression by an RNA interference approach increased the sensitivity of PDAC cells to gemcitabine [50]. In addition to primary chemotherapy resistance, MYC has been shown to be involved in secondary resistance to paclitaxel [51]. Low-passaged primary PDAC cultures and a ramp-up protocol to induce paclitaxel resistance were used to generate secondary resistant models. Analysis of the models illustrated increased expression of MYC mRNA and protein expression. Furthermore, reduction in MYC expression increased paclitaxel-induced cell death in the resistant lines, whereas overexpression reduced paclitaxel-induced cell death in the parental PDAC cells [51]. Interestingly, gemcitabine was found to trigger a MYC-associated vulnerability in unbiased screening experiments [38, 52, 53] and deregulated MYC sensitizes to mitotic perturbants, including paclitaxel [54, 55]. Such data highlight the need for further research on MYC-associated vulnerabilities and point to a specific context, exemplified here by lineage or secondary versus primary resistance, that needs to be understood.

## Targeting MYC in PDAC

**Fig. 1:**
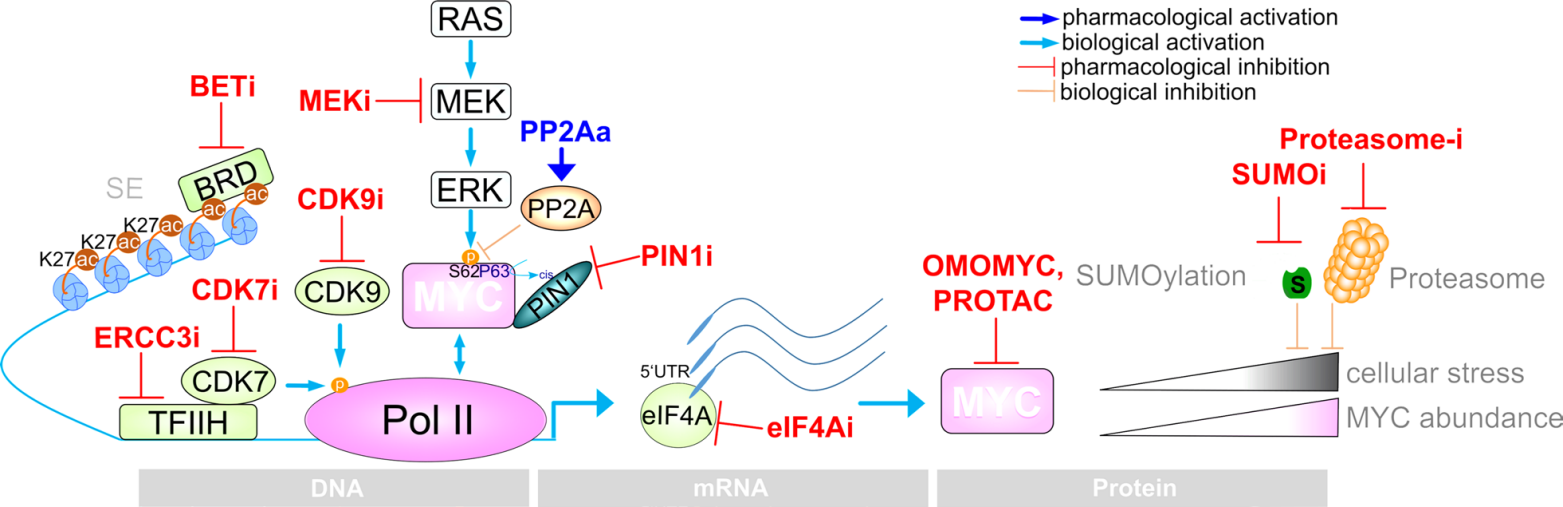
Targeting of MYC. Illustrated are options of direct or indirect MYC inhibition by pharmacological inhibitors or activators, respectively. Indirect inhibition is achieved by interfering with transcription, translation. The concept to target MYC by synthetic lethality in PDAC is shown. Direct inhibition by a synthetic OMOMYC peptide or possibly by PROTACs renders the MYC protein itself nonfunctional. *SE* Super enhancer, *BRD* Bromodomain proteins

Direct targeting of MYC remains a challenge and no small molecule inhibitor has made it to the clinic so far [13, 56]. Due to recent developments, we will only shortly describe OMOMYC, a dominant negative MYC dimerization inhibitor. OMOMYC, a 91 amino acids mutant version of the MYC dimerization domain, prevents MYC from binding to its target genes. In several cancer mouse models, conditional expression of OMOMYC dramatically impacts on tumor growth [17, 57, 58]. In vitro, an inducible OMOMYC reduced the clonogenic growth of murine PDAC cell lines [59]. The concept was recently advanced by the development of an OMOMYC mini-protein. The in vivo efficacy of the mini-protein was demonstrated in non-small cell lung cancer (NSCLC) models [18]. The mini-protein penetrates many organs, including the pancreas [18]. Importantly, a phase I/II clinical trial of the OMOMYC mini-protein (OMO-103) was started in 2021 (NCT04808362), bringing direct MYC inhibition to the clinic. In addition to OMOMYC mini-proteins, it is foreseeable that advanced drug development methods, such as the proteolysis targeting chimera (PROTAC) technology, will lead to the development of clinical candidates that directly and specifically targeting MYC (Fig. 1) [60].

Several approaches to indirectly target MYC expression exist, and we will summarize some with documented relevance in PDAC (Fig. 1). Members of the bromo- and extra-terminal domain (BET) motif protein family, BRD2, BRD3, BRD4, and BRDT, are involved in the regulation of many cancer-relevant pathways, and BET inhibitors (BETi) are in clinical development [61]. Expression of cancer driver genes is often regulated by so-called super-enhancers (SE), specialized *cis*-acting regulatory elements characterized by an enriched acetylation of histone H3 lysine 27 (H3K27ac), which stimulate high-level expression of associated genes. These enhancers are particularly sensitive to BETi [62] so that oncogenic drivers, including MYC, can be inhibited. Inhibition of MYC expression by BETi has also been documented in PDAC [63]. In primary patient-derived xenografts, a less differentiated PDAC subtype, with a high proliferation index and shorter survival, was characterized [64]. This subtype was characterized by a MYC-driven transcriptional program and sensitivity to BETi [64]. The study was recapitulated in human PDAC organoids, a model with a potential predictive power for the clinical behavior of solid cancers [65]. Again, the defined MYC-signature co-segregates with higher sensitivity to the BETi JQ1 and NHWD-870 [66].

In addition to BETi, inhibitors of histone deacetylases (HDAC) target MYC expression and the MYC-driven transcriptional program in PDAC [67]. Furthermore, HDAC were shown to tune the MYC program and repress a gene program linked to epithelial differentiation [68]. Blocking HDAC function by inhibitors induces these genes in a BRD4-dependent fashion [68], furthermore underlining the close interplay between HDAC and BET proteins. Such connections might contribute to the described synergism of BETi and HDACi in patient-derived xenograft models and are underpinned by the profound effect of the combined inhibition of HDACs and BET on MYC protein expression [63]. Recently dual BET/HDAC inhibitors were developed and tested in PDAC models. Notably, after prolonged treatment, TW9, a dual BET/HDACi, distinctly reduced MYC expression [69]. However, other SE-controlled cancer drivers, including the AP1-TF family member *FOSL1*, also appear to be targeted by TW9 [69].

The ATP-dependent DNA helicase excision repair cross-complementation protein 3 (ERCC3) is a component of the general transcription factor TFIIH, which is part of the transcriptional preinitiation complex (Fig. 1). ERCC3 can be targeted by the natural diterpenoid epoxide triptolide [70]. In an unbiased pharmacological screening experiment, high activity of triptolide for PDAC was found. Mechanistically, triptolide reduced *MYC* mRNA and protein expression [71] by disrupting the activity of SE and causing downregulation of associated genes, including *MYC* [72]. The high efficacy of triptolide has been demonstrated in in vitro and in vivo models of PDAC with deregulated MYC expression [71], allowing to tackle this particular subtype.

In addition to transcriptional or post-translational interference with MYC, the translation of MYC can also be pharmacologically disrupted (Fig. 1). The mRNA of *MYC* harbors a structured 5′UTR, which is characteristic for translation controlled by the RNA helicase eukaryotic initiation factor 4A (eIF4A) [73]. Therefore, the use of eIF4A inhibitors to impair MYC expression in PDAC is an attractive option. The eIF4A inhibitor CR-1-31B demonstrated high efficacy both in vivo and in vitro [73, 74]. This class of inhibitors (e.g., zotatifine) is currently being tested in a phase 1–2 clinical trial in PDAC patients (NCT04092673). Consistent with the complex cross-talk of MYC with the TME, the natural eIF4A inhibitor silvestrol augmented the activity of an anti-PD1 antibody therapy [75].

The peptidyl-prolyl cis–trans isomerase NIMA-interacting 1 (PIN1), which induces conformational changes in certain phosphorylated proteins, is overexpressed in many cancers [76]. Proline-guided serine or threonine phosphorylation is a typical PTM, conducted by kinases like ERK or cyclin-dependent kinases (CDKs). PIN1 complexes with serine 62-phosphorylated MYC leading to trans > cis isomerization of proline 63 [77], which prevents phosphatase activity and an increased DNA binding capacity of MYC. Genetic gain- and loss-of-function experiments have demonstrated that the transcriptional output of MYC is controlled by PIN1 [78]. Recently the covalent, highly selective PIN1 inhibitor sulfopin was shown to downregulate the MYC transcriptional network [79]. Interestingly, sulfopin reduced PDAC growth and prolonged survival in an immune-proficient orthotopic transplantation model in vivo [79].

## Targeting MYC-associated vulnerabilities

MYC renders cancer cells dependent on a properly functioning transcriptional machinery [80]. Initiation, pausing, and elongation of the RNA polymerase II (Pol II) are tightly controlled. One control mechanism is integrated by phosphorylation of the carboxy-terminal domain (CTD) of the largest subunit of Pol II, with the CTD integrating the activity of the transcriptional CDKs, like CDK7 or CDK9 [81]. CDK7 acts at multiple sites in the transcriptional cycle and is involved in Pol II-dependent transcriptional entry as well as in the activation of CDK9, involved in transcriptional pause release [81]. A pharmacological screening of epigenetic drugs in human PDAC cell lines revealed the CDK7i THZ1 as a prominent hit. The sensitivity of THZ1 strongly correlated with MYC expression and the activity of the associated network [82]. Selective CDK7 inhibitors, such as the orally available CDK7i CT7001 or SY-5609, are currently tested in clinical trials in patients with solid cancers (NCT03363893, NCT04247126), demonstrating the clinical potential. In addition to CDK7, higher activity of CDK9i in cancers with deregulated MYC is already known [83]. In PDAC, the connection of CDK9i (UNC10112785) to MYC was recently found in a screen using a MYC degradation reporter system. In addition to the decrease in *MYC* mRNA, CDK9 was shown to induce phosphorylation of serine 62 and thus MYC stability, arguing for a feed forward loop [84].

A drug screening of FDA approved anti-cancer drugs and analysis of several public repositories revealed an increased sensitivity toward perturbations of the protein homeostasis in MYC^high^ PDAC cells [52]. MYC orchestrates a variety of biological processes by regulating and tuning the transcriptional and translational output [85]. Due to the high amount of protein load in cancer cells and a protein biosynthesis machinery acting at the upper limit, cells with high MYC expression are associated with an increased unfolded protein response (UPR). The strong connection of MYC activity and the UPR is also documented across species [86] and cancer entities [87, 88], explaining increased sensitivity to perturbations in protein homeostasis.

A recent study connected MYC to PDAC with increased expression of the small ubiquitin-like modifier (SUMO)-ylation machinery [89]. The enzymatic cascade of SUMOylation is analogous to ubiquitination, which consists of a single heterodimeric SUMO activating enzyme (E1), a single SUMO conjugating enzyme (E2), and less well-defined SUMO ligases (E3) [21, 88]. Recently, pharmacological SUMO inhibition was shown to be particularly potent in PDAC cells, which are characterized by high MYC activity [89]. This synthetic lethality has already been demonstrated in other entities [90–92] and demonstrated a MYC-dependent and entity-independent vulnerability. SUMO inhibitors such as Subasumstat (TAK-981) are currently under clinical development in advanced solid cancers (NCT04381650).

## Imaging MYC in PDAC

**Fig. 2:**
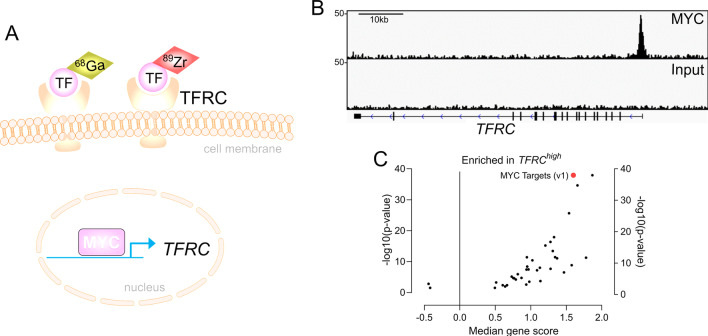
Imaging of MYC. **A** Scheme for non-invasive annotating the MYC status. The *TFRC* gene is a MYC target. The Transferrin receptor can be imaged with a radiotracer, here ^89^Zr-labeled transferrin (TF) or ^68^Ga-citrate, which binds transferrin (TF). Overexpression of MYC leads to increased TFRC expression and augmented PET signal. **B** MYC and control Input ChIP-Seq data of human PDAC cells (MiaPaCa-2) published by Bhattacharyya et al. 2020 [36], were analyzed for specific binding of MYC to the *TFRC* gene. In addition, the exon structure of the *TFRC* gene is depicted (black boxes). **C** A mRNA expression dataset of human PDAC were retrieved via the publication of Bailey et al. [22] and curated as described [52]. A gene set enrichment analysis (GSEA) of Hallmark signatures via the GeneTrial 3.0 platform [110] was conducted comparing PDACs with *TFRC* mRNA expression in the highest quartile (Q4) versus PDACs in the quartiles Q1, Q2, and Q3. Each dot represents a significant hallmark signature. The MYC HALLMARK (v1) signature is highlighted in red

Considering the strong association of MYC with drug responsive states, non-invasive imaging-based biomarkers indicative for activity of MYC will help to stratify PDAC patients for specific therapies. Nearly three decades ago, Indium-111-labeled antisense-oligonucleotides were used to measure *MYC* mRNA in breast cancer mouse models [93]. However, this imaging method revealed hurdles, such as limited tracer delivery due to physical barriers, target sequence selection, or low stability [94]. In 2012, the radiotracer ^89^Zirconium (Zr)-desferrioxamine transferrin (^89^Zr-Tf) was developed and ^89^Zr-Tf PET imaging was shown to annotate the MYC status [95]. The transferrin receptor (TfR1, TFRC, CD71; TFRC afterwards) is a transmembrane glycoprotein, which acts as a disulfide bond-linked dimer. Transferrin, bound by two iron atoms, shows the highest affinity for the TFRC, and upon binding, transferrin and the receptor are internalized by endocytosis and iron replenish the intracellular pools [96]. Iron is needed for many cellular processes serving the demands of cancer cells, including mitochondrial respiration to generate energy, building of ribonucleotides for DNA synthesis, or DNA repair [96]. MYC directly activates the *TFRC* gene (Fig. 2) to serve the iron demands of aggressive cancers [97], allowing to indirectly image its activity. Initially ^89^Zr-Tf PET imaging was developed to annotate MYC in prostate cancer models [95]. Meanwhile, this imaging modality was demonstrated to define the MYC status in other tumor entities, like triple negative breast cancer models [98] and allows monitoring of treatment induced changes of MYC expression (Fig. 2) [99]. ^89^Zr-Tf PET imaging was also evaluated in PDAC models and determines the extent of the KRAS signaling output and the downstream integrator MYC [100]. Furthermore, the imaging modality was shown to be useful to monitor pharmacological interference with pathways integrated by MYC or drugs which target MYC [100].

Alternatives to the ^89^Zr-Tf PET imaging were also developed. A human anti-transferrin receptor monoclonal antibody labeled with ^89^Zr as a PET probe was investigated in PDAC and shown to allow measurement of the TFRC in xenograft models [101]. In addition, ^68^Ga-citrate, which binds to transferrin, can be used to image the TFRC (Fig. 2) [102]. In prostate cancer models, PET imaging with the tracer ^68^Ga-citrate was shown to substitute the ^89^Zr-Tf PET imaging and some hints of the association of the signal with MYC deregulated cancers were provided [103], increasing the number of imaging modalities to annotate MYC. However, a combination of different imaging and genetic approaches may allow more precise stratification and further prospective studies are needed to validate MYC centric imaging modalities.

## Conclusions

Successful concepts for precision oncology require strong drivers of cancer subtypes as biomarkers, that are associated with different drug sensitivity phenotypes, like MYC. However, multiple layers of such an approach need to be advanced, from a better understanding of the MYC-associated drug response to modalities for mapping MYC activity.

Current markers of drug responsiveness only enrich for a responding population, and even cancer stratified by MYC will only increase the proportion of patients who respond to a particular therapy. Therefore, the drug response in PDACs with high MYC activity that does not respond to therapies and trigger associated vulnerability needs to be better characterized and will lead to bi- or trivalent selection markers and molecular combination therapies. Therefore, the context in which MYC operates needs to be better defined experimentally.

Molecular and functional imaging also needs to evolve, and the integration of multimodal imaging can increase the precision in terms of biomarkers and targets. Since MYC is a central regulator of glycolysis and regulates the transcription of glycolytic genes, such as *HK2*, *ENO1*, *LDHA*, *SLC2A1* [104, 105], glycolysis might be used as surrogate and an indirect imaging approach to determine MYC activity. In a MYC-inducible model of liver cancer, glycolysis could be visualized by hyperpolarized ^13^C magnetic resonance spectroscopy and its activity could be attributed to the MYC oncogene [106]. In breast cancer, ^18^F-FDG PET can image basal-like cancers with deregulated MYC [107]. Glycolysis- and MYC-networks enrich in basal-like PDAC [11], offering an additional opportunity for imaging. As a future example of a multimodal imaging approach, ^18^F-FDG PET combined with imaging of the MYC-TFRC circuit might enable a more accurate discrimination of basal-like subtypes with deregulated MYC. However, other oncogenic pathways, particularly the PI3K-AKT pathway [108] or hypoxia-mediated activation of the hypoxia-inducible factor 1 (HIF) [109], have also been shown to induce glycolysis. Therefore, there is a definite need for more pre-clinical evidence of such concepts followed by prospective clinical evaluation. In addition, such clinical non-invasive imaging modalities demand to be accompanied by longitudinal biopsy programs to link imaging with molecular features. Finally, more direct non-invasive MYC imaging modalities should be developed. Interestingly, the OMOMYC mini-protein labeled with ^89^Zr (Omomyc-deferoxamin-maleimide(DFO)-^89^Zr) was shown to accumulate in mouse lung tumors after intranasal application [18], which will allow further development of OMOMYC mini-proteins into imaging probes for annotation of MYC status as well as for cancer theranostics.

## Data Availability

Not applicable.
